# Infections and severe mental illness: a population-based matched cohort study

**DOI:** 10.1136/bmjment-2026-302640

**Published:** 2026-05-27

**Authors:** Sharon L Cadogan, Georgia R Gore-Langton, Kathryn E Mansfield, John Tazare, Seena Fazel, Ian J Douglas, Caroline Morton, Naaheed Mukadam, Charlotte Warren-Gash

**Affiliations:** 1 Faculty of Epidemiology and Population Health, London School of Hygiene and Tropical Medicine, London, UK; 2 School of Health and Care Sciences, University of Lincoln, Lincoln, UK; 3 Department of Psychiatry, University of Oxford, Oxford, UK; 4 Division of Psychiatry, University College London, London, UK

**Keywords:** Bipolar and Related Disorders, Schizophrenia, Schizophrenia Spectrum and Other Psychotic Disorders

## Abstract

**Background:**

It is unclear whether acute infections associated with short-term systemic inflammation influence the risk of severe mental illness (SMI).

**Objective:**

To investigate relationships between acute infections and incident SMI using electronic health records from the UK.

**Methods:**

Using data from the Clinical Practice Research Datalink Aurum (1 January 2007 to 15 June 2024), we conducted six matched cohort studies. Adults (≥18 years) with gastroenteritis (GE), lower respiratory tract infection (LRTI), skin and soft tissue infection (SSTI), urinary tract infection (UTI), sepsis and meningitis/encephalitis (positive control exposure) recorded in primary care were matched with up to five individuals without infection on age, sex and practice in calendar date order and followed for incident SMI (schizophrenia, bipolar disorder, other psychoses). We estimated HRs for SMI comparing those with and without each infection using Cox regression, stratified by match set, adjusting for potential confounders (deprivation, Charlson Comorbidity Index, alcohol, smoking, body mass index and ethnicity).

**Findings:**

Our six study cohorts ranged from 2 089 168 adults (391 773 with SSTI, 1 697 395 without) to 106 155 (17 860 with meningitis/encephalitis, 88 295 without). Median follow-up ranged from 4.1 years (IQR 1.9–6.9) in the sepsis cohort to 5.7 years (IQR 2.6–9.8) in the meningitis/encephalitis cohort. After adjustment, each infection was associated with increased SMI risk: SSTI, HR 1.16 (95% CI 1.08 to 1.24); LRTI, 1.28 (95% CI 1.20 to 1.38); UTI, 1.44 (95% CI 1.31 to 1.58); GE, 1.53 (95% CI 1.42 to 1.65); sepsis, 1.69 (95% CI 1.52 to 1.88); and meningitis/encephalitis, 3.36 (95% CI 2.61 to 4.32).

**Conclusion:**

Our findings suggest SMI risk is higher among adults with a range of acute infections compared with those without, with higher risks for the more severe infections meningitis/encephalitis and sepsis.

**Clinical implications:**

Providing timely infection treatment, targeted mental health support following severe infections, and where appropriate offering relevant vaccinations, may limit SMI risk.

**WHAT IS ALREADY KNOWN ON THIS TOPIC**

- People with central nervous system infections have an increased risk of developing severe mental illness (SMI). It is not known whether acute infections presenting to primary care are associated with increased SMI risk.

**WHAT THIS STUDY ADDS**

- Using a series of infection-specific matched cohorts defined in electronic health records, each infection type (sepsis, lower respiratory tract infection, gastroenteritis, urinary tract infection, skin and soft tissue infection, and encephalitis/meningitis (positive control exposure)) was associated with higher SMI risk after controlling for sociodemographic, lifestyle, and clinical confounders.

**HOW THIS STUDY MIGHT AFFECT RESEARCH, PRACTICE OR POLICY**

- Our study identified increased SMI risks after infections affecting a range of sites. Optimising infection prevention, providing timely infection treatment and targeted mental health risk management following severe infections may reduce incident SMI.

## Background

 Severe mental illness (SMI) (ie, schizophrenia, bipolar disorder, other psychoses) is a major public health concern in the UK and worldwide. Globally, people with SMI die 10–20 years earlier than the general population^1^,^3^ and emerging evidence suggests this mortality gap may be widening.^4^ Infections have increasingly been recognised as potential triggers, or exacerbators, of mental health conditions, contributing to substantial morbidity and mortality.^5 6^ We have recently reported an association between common, acute infections and an elevated risk of anxiety and depression.^7^ In this study, we investigate infections and associated SMI risk.

As peak incidence of both schizophrenia and bipolar disorder is typically in late adolescence or early adulthood, existing research has tended to focus on early life risk factors. Systematic reviews suggest that severe infections in childhood requiring hospitalisation are associated with an increased risk of schizophrenia (OR 1.44, 95% CI 1.19 to 1.73) and psychosis (OR 1.27, 95% CI 1.13 to 1.44), rising to OR 1.68 (95% CI 1.08 to 2.62) for the effect of central nervous system (CNS) infections alone on future psychosis risk.^8^ Psychosis risk is also elevated among children who experience less severe infections requiring outpatient care only.^9^ Proposed mechanisms include through dysregulated neuroimmune pathways with infection-triggered systemic and CNS inflammation disrupting brain development and function.^10^

Evidence is more limited for infections occurring in adults. Among individuals with a median age of 45 years, a matched cohort study from the UK showed that encephalitis was associated with a range of psychiatric sequelae including bipolar affective disorder (adjusted RR 6.34, 95% CI 3.34 to 12.04) and psychotic disorders 3.48 (95% CI 2.18 to 5.57).^11^ One study of severe infections requiring hospitalisation showed increased schizophrenia risk following infections experienced in early adulthood, for example, adjusted RR 1.73 (95% CI 1.41 to 2.11) for individuals aged 20–24 years.^12^ While a range of mental health conditions have been linked to pandemic influenza^13^ and COVID-19,^14^ disaggregating effects of acute infection from pandemic mitigation measures is challenging.^15^

Overall, it is unclear whether common, acute non-CNS infections occurring in adults, especially those that do not require critical care or hospital treatment have any associations with subsequent SMI. To address this, we used routinely collected primary care data from the Clinical Practice Research Datalink (CPRD) Aurum, and linked data, in a series of matched cohort studies to compare SMI risk in people with and without a primary care diagnosis of six acute infections (gastroenteritis (GE), lower respiratory tract infection (LRTI), skin and soft tissue infection (SSTI), urinary tract infection (UTI), sepsis or, as a positive control exposure, meningitis/encephalitis).

## Methods

### Ethics approval

Consent is given by the primary care practices contributing data to CPRD. Individual patient consent is implied; however, patients are offered the right to opt out from the use of their pseudo-anonymised data.

### Data source

We used primary care electronic health record data from the CPRD Aurum (August 2024 build), a large primary care database of routinely collected, pseudo-anonymised medical records covering over 19 million patients.^16^ Aurum contains information on diagnoses, symptoms,and prescriptions recorded by healthcare professionals from practices predominantly based in England, with some in Northern Ireland. We also used linked data from: (1) Hospital admission data for English National Health Service-funded patients (Hospital Episode Statistics (HES)); (2) Mortality data from the Office of National Statistics (ONS); and (3) individual-level Index of Multiple Deprivation (IMD) data^17^ (latest release 2019, supplemented with practice level IMD, where individual-level data were not available).

### Study design and population

We conducted six matched cohort studies, one for each infection exposure; GE, LRTI, SSTI, UTI, sepsis, and meningitis/encephalitis (as a positive control exposure). We limited to individuals aged 18 and over, with at least 1 year of prior registration with a CPRD Aurum practice between 1 January 2007 and 15 June 2024. Individuals were excluded if they had a record of SMI prior to infection (online supplemental figure 1). Due to large numbers of individuals with the four most common infections (ie, GE, LRTI, SSTI and UTI) we took a random 10% sample of all individuals with a primary care record of each of these infections. In the sepsis and meningitis/encephalitis cohorts, all individuals with sepsis or meningitis/encephalitis, as appropriate to the exposure cohort, were included (online supplemental methods 1).

### Exposure and follow-up

Individuals with infections (GE, LRTI, SSTI, UTI, sepsis, meningitis/encephalitis) entered the infection-specific cohort on the date of their first record for the specific infection (index date). Individuals were defined as having each infection if they had at least one record of that infection during the study period. While individuals could appear in multiple cohorts, for example, if they had a record of GE and SSTI, the infection cohorts were considered separately. We randomly matched without replacement, each individual with infection with up to five people without that infection on age (within 2 years), sex, and primary care practice in calendar data order (ie, the matching algorithm assigned matched comparators first to individuals with infection with earliest cohort entry to avoid time-related bias).^18^ Matched individuals without infection (comparison cohort) entered their respective cohorts on the index date of their matched individual with infection. We only retained matched sets including at least one individual with infection and one without.

Individuals were followed until the earliest of: (1) first SMI diagnosis; (2) death recorded in CPRD or ONS; (3) end of registration with practice; (4) no further data collected from practice; or (5) study end (15 June 2024). Individuals in the comparator cohorts who subsequently received a diagnostic code for the specific infection after cohort entry were censored from the comparison group and became eligible for inclusion in the infection-exposed group. Individuals with 1 day of follow-up were excluded.

### Outcome

We defined SMI based on the earliest record of an incident SMI diagnostic code (bipolar, schizophrenia, or other psychosis code) recorded in primary care (CPRD). Individuals were censored when there was a record for psychosis with a clear underlying cause (ie, organic psychosis) such as drug-induced psychosis, which was unlikely to be influenced by infections.

### Covariates

We considered the following as potential confounders of the relationship between infection and SMI: matching factors (ie, age, sex (male, female), general practice), chronic comorbidities, ethnicity, lifestyle factors (smoking, harmful alcohol intake, obesity) and deprivation. Code lists used to define all study variables are available to download: https://datacompass.lshtm.ac.uk/id/eprint/4923/. Further details of study variable definitions are in online supplemental table 1.

### Statistical analysis

We described the characteristics (including age, sex, deprivation, lifestyle factors, ethnicity, chronic comorbidities) of the study population at cohort entry by infection status. We also calculated age- and sex-adjusted SMI incidence rates in those with and without the relevant infection.

#### Main analysis

We used Cox regression, stratified by matched set, and adjusted for potential confounders to estimate HRs and corresponding 95% CIs for the association between each infection and SMI. We used sequential adjustment models, initially examining a crude model and implicitly adjusting for the matching variables (age, sex and general practice, and calendar time) by stratifying by matched set (Model 1). We then additionally adjusted for other potential explanatory variables: Model 2, adjusted for deprivation (quintiles of IMD); Model 3, further adjusted for smoking status, alcohol consumption, obesity and comorbidities (using the Charlson Comorbidity Index (CCI)); and Model 4, additionally adjusted for ethnicity (adjusted for last to limit selection bias due to relatively high proportion of missing ethnicity data).

We used Stata MP statistical software V.19 (StataCorp) for all analyses. The programming code used for data management and statistical analysis is available at: 10.5281/zenodo.18497941

#### Sensitivity analyses

We conducted a series of sensitivity analyses to assess the robustness of our findings: (1) restricting the analysis to individuals with at least one primary care consultation in the year prior to cohort entry (to exclude non-attenders); (2) repeating the main analysis without censoring at organic psychosis diagnoses to avoid missing outcomes; (3) repeating the main analyses with start of follow-up 1 year after infection and excluding individuals with SMI diagnoses in the year between index date and start of follow-up to limit outcome ascertainment bias or reverse causality; (4) in the SSTI cohort only, additionally adjusting for recreational injectable drug use (based on morbidity coding indicating recreational injectable drug) which predisposes SSTI and SMI; (5) repeating our analysis with a revised end date of 1 March 2020 to explore the impact of including pandemic time in our analyses; and (6) repeating main analysis using a missing category approach for ethnicity, due to a high proportion of missing data.

#### Secondary analyses

In prespecified secondary analyses, we assessed whether the association between infections and SMI: (1) persisted or waned over time by repeating our main analyses in different time periods following infection (0–6 months; 0–1 year; 0–2 years; 0–3 years; 0–4 years; 0–5 years); (2) differed by GE, LRTI, SSTI and UTI infection severity (sepsis and meningitis/encephalitis were assumed to be severe). To do this we restricted to individuals eligible for HES linkage and categorised infections as ‘severe’ if there was a record for a hospital admission for the same infection or sepsis (recorded using International Classification of Diseases, Version 10 morbidity codes in any diagnostic position of any episode of a hospital admission) 28 days either side of the primary care record, or ‘not severe’ if there was only a primary care record; (3) differed depending on whether or not GE, LRTI, SSTI or UTI infections had a corresponding antimicrobial prescription within 7 days of infection diagnosis. Prescriptions included were for the following drug classes: penicillin, fluoroquinolones, macrolides and cephalosporins plus drugs listed in the NICE (National Institute for Health and Care Excellence) British National Formulary treatment summary for UTIs; and (4) was modified by age at infection, sex or frailty (restricting to those 65–95 years, the population in which the electronic frailty index was validated) to investigate potential high risk groups.^12^

## Results

Our six infection-specific matched cohorts ranged in size from 106 155 adults (17 860 with meningitis/encephalitis, 88 295 without) in the meningitis/encephalitis cohort, table 1, to 2 089 168 adults (391 773 with SSTI, 1 697 395 without) in the SSTI cohort, table 2 and online supplemental figure 2. Median follow-up ranged from 4.1 years (IQR 1.9–6.9) in the sepsis cohort, to 5.7 years (IQR 2.6–9.8) in the meningitis/encephalitis cohort (table 1). Median follow-up in most infection cohorts was around 5 years.

**Table 1:** Characteristics of study participants at cohort entry, by infection cohort and infection status, for sepsis and meningitis/encephalitis cohorts

Infection status	Sepsis (N=1 094 657)		Meningitis/encephalitis (N=106 155)	
	With infection	Without infection	With infection	Without infection
Total	185 568 (17.0)	909 089 (83.0)	17 860 (16.8)	88 295 (83.2)
Median (IQR) follow-up years	2.5 (0.7–5.5)	4.4 (2.2–7.1)	5.3 (2.2–9.6)	5.8 (2.6–9.9)
Median age (IQR)	73 (59–83)	73 (59–82)	42 (30–60)	42 (30–60)
Median (IQR) consultations in year before index*	34 (22–50)	15 (7–26)	16 (8–28)	8 (3–17)
Age				
18–29	6781 (3.7)	33 701 (3.7)	4257 (23.8)	21 175 (24.0)
30–39	8619 (4.6)	42 618 (4.7)	3830 (21.4)	18 953 (21.5)
40–59	31 480 (17.0)	154 947 (17.0)	5234 (29.3)	25 779 (29.2)
60+	138 688 (74.7)	677 823 (74.6)	4539 (25.4)	22 388 (25.4)
Sex				
Female	90 632 (48.8)	444 237 (48.9)	9985 (55.9)	49 387 (55.9)
Male	94 936 (51.2)	464 852 (51.1)	7875 (44.1)	38 908 (44.1)
Ethnicity				
Black	2538 (1.4)	12 854 (1.4)	437 (2.4)	1706 (1.9)
Mixed other	28 640 (15.4)	150 109 (16.5)	2849 (16.0)	13 694 (15.5)
South Asian	5726 (3.1)	27 008 (3.0)	743 (4.2)	3731 (4.2)
White	120 549 (65.0)	610 632 (67.2)	11 236 (62.9)	54 425 (61.6)
Unknown	28 115 (15.2)	108 486 (11.9)	2595 (14.5)	14 739 (16.7)
Smoking status				
People who smoke currently or people who have quit smoking	97 104 (52.3)	432 014 (47.5)	8400 (47.0)	38 126 (43.2)
People who do not smoke	87 685 (47.3)	465 494 (51.2)	9243 (51.8)	47 290 (53.6)
Missing	779 (0.4)	11 581 (1.3)	217 (1.2)	2879 (3.3)
Obese				
Not obese (BMI <30.0 kg/m²)	122 892 (66.2)	645 518 (71.0)	12 124 (67.9)	59 909 (67.9)
Obese (BMI ≥30.0 kg/m²)	53 790 (29.0)	215 292 (23.7)	4392 (24.6)	18 181 (20.6)
Missing	8886 (4.8)	48 279 (5.3)	1344 (7.5)	10 205 (11.6)
Charlson Comorbidity Index				
Low (0)	29 399 (15.8)	361 723 (39.8)	9802 (54.9)	58 972 (66.8)
Moderate (1–2)	59 150 (31.9)	294 533 (32.4)	5448 (30.5)	21 892 (24.8)
Severe (3 or more)	97 019 (52.3)	252 833 (27.8)	2610 (14.6)	7431 (8.4)
IMD quintile				
1, least deprived	34 571 (18.6)	186 310 (20.5)	3536 (19.8)	18 227 (20.6)
2	34 604 (18.6)	179 587 (19.8)	3431 (19.2)	16 762 (19.0)
3	34 554 (18.6)	170 211 (18.7)	3342 (18.7)	16 320 (18.5)
4	40 258 (21.7)	188 916 (20.8)	3779 (21.2)	18 683 (21.2)
5, most deprived	41 015 (22.1)	181 310 (19.9)	3713 (20.8)	18 010 (20.4)
Missing	566 (0.3)	2755 (0.3)	59 (0.3)	293 (0.3)

Data are n (column %) unless otherwise specified.

* GP consultations in the year before cohort entry.

BMI, body mass index; IMD, Index of Multiple Deprivation.

**Table 2:** Characteristics of study participants at cohort entry, by infection cohort and infection status, for gastroenteritis, lower respiratory tract infection, skin and soft tissue infection, and urinary tract infection cohorts

Infection status	Gastroenteritis (N=1 672 843)		Lower respiratory tract infection (N=1 954 736)		Skin and soft tissue infection (N=2 089 168)		Urinary tract infection (N=1 040 648)	
	With infection	Without infection	With infection	Without infection	With infection	Without infection	With infection	Without infection
Total	306 455 (18.3)	1 366 388 (81.7)	373 286 (19.1)	1 581 450 (80.9)	391 773 (18.8)	1 697 395 (81.2)	189 389 (18.2)	851 259 (81.8)
Median (IQR) follow-up years	6.1 (2.6–10.9)	5.2 (2.2–9.7)	6.5 (2.7–11.4)	5.2 (2.1–9.5)	6.6 (3.0–11.2)	5.0 (2.2–9.3)	5.9 (2.4–10.7)	5.3 (2.3–9.8)
Median age (IQR)	51 (33–70)	50 (33–68)	58 (41–73)	56 (40–71)	51 (35–68)	50 (34–67)	56 (35–74)	55 (35–73)
Median (IQR) consultations in year before index*	16 (8–28)	8 (3–17)	16 (8–27)	9 (3–18)	14 (7–25)	8 (3–17)	18 (10–30)	10 (4–19)
Age								
18–29	57 942 (18.9)	271 385 (19.9)	38 165 (10.2)	180 874 (11.4)	64 682 (16.5)	300 929 (17.7)	32 604 (17.2)	152 565 (17.9)
30–39	46 069 (15.0)	211 721 (15.5)	46 100 (12.3)	211 337 (13.4)	58 414 (14.9)	264 822 (15.6)	24 496 (12.9)	113 440 (13.3)
40–59	83 862 (27.4)	379 551 (27.8)	114 057 (30.6)	499 489 (31.6)	120 838 (30.8)	528 832 (31.2)	45 501 (24.0)	208 718 (24.5)
60+	118 582 (38.7)	503 731 (36.9)	174 964 (46.9)	689 750 (43.6)	147 839 (37.7)	602 812 (35.5)	86 788 (45.8)	376 536 (44.2)
Sex								
Female	177 285 (57.9)	781 854 (57.2)	211 074 (56.5)	885 172 (56.0)	220 011 (56.2)	943 041 (55.6)	151 573 (80.0)	674 823 (79.3)
Male	129 170 (42.1)	584 534 (42.8)	162 212 (43.5)	696 278 (44.0)	171 762 (43.8)	754 354 (44.4)	37 816 (20.0)	176 436 (20.7)
Ethnicity								
Black	5524 (1.8)	27 452 (2.0)	4766 (1.3)	27 208 (1.7)	6313 (1.6)	32 193 (1.9)	2578 (1.4)	15 352 (1.8)
Mixed other	49 352 (16.1)	210 841 (15.4)	58 979 (15.8)	237 722 (15.0)	61 705 (15.8)	260 263 (15.3)	29 495 (15.6)	131 797 (15.5)
South Asian	13 602 (4.4)	56 532 (4.1)	14 575 (3.9)	57 561 (3.6)	15 430 (3.9)	69 825 (4.1)	6632 (3.5)	31 481 (3.7)
White	192 598 (62.8)	830 734 (60.8)	242 529 (65.0)	986 292 (62.4)	253 844 (64.8)	1 043 607 (61.5)	122 446 (64.7)	529 860 (62.2)
Unknown	45 379 (14.8)	240 829 (17.6)	52 437 (14.0)	272 667 (17.2)	54 481 (13.9)	291 507 (17.2)	28 238 (14.9)	142 769 (16.8)
Smoking status								
Current- or ex-smoker	144 898 (47.3)	592 868 (43.4)	194 727 (52.2)	695 599 (44.0)	187 834 (47.9)	737 644 (43.5)	82 685 (43.7)	355 259 (41.7)
Non-smoker	159 239 (52.0)	732 468 (53.6)	176 968 (47.4)	845 724 (53.5)	200 474 (51.2)	907 802 (53.5)	105 912 (55.9)	479 752 (56.4)
Missing	2318 (0.8)	41 052 (3.0)	1591 (0.4)	40 127 (2.5)	3465 (0.9)	51 949 (3.1)	792 (0.4)	16 248 (1.9)
Obese								
Not obese (BMI <30.0 kg/m²)	210 808 (68.8)	941 069 (68.9)	248 924 (66.7)	1 098 276 (69.4)	257 023 (65.6)	1 175 117 (69.2)	135 821 (71.7)	603 582 (70.9)
Obese (BMI ≥30.0 kg/m²)	75 629 (24.7)	279 051 (20.4)	104 418 (28.0)	337 259 (21.3)	108 914 (27.8)	342 227 (20.2)	44 104 (23.3)	183 157 (21.5)
Missing	20 018 (6.5)	146 268 (10.7)	19 944 (5.3)	145 915 (9.2)	25 836 (6.6)	180 051 (10.6)	9464 (5.0)	64 520 (7.6)
Charlson Comorbidity Index								
Low (0)	156 682 (51.1)	858 575 (62.8)	164 291 (44.0)	962 065 (60.8)	209 712 (53.5)	1 080 729 (63.7)	92 673 (48.9)	499 532 (58.7)
Moderate (1–2)	94 303 (30.8)	355 306 (26.0)	134 203 (36.0)	424 432 (26.8)	120 011 (30.6)	438 261 (25.8)	57 764 (30.5)	235 117 (27.6)
Severe (3 or more)	55 470 (18.1)	152 507 (11.2)	74 792 (20.0)	194 953 (12.3)	62 050 (15.8)	178 405 (10.5)	38 952 (20.6)	116 610 (13.7)
IMD quintile								
1, least deprived	61 006 (19.9)	280 540 (20.5)	71 851 (19.2)	325 446 (20.6)	81 334 (20.8)	359 291 (21.2)	39 718 (21.0)	181 462 (21.3)
2	56 763 (18.5)	260 373 (19.1)	69 737 (18.7)	307 499 (19.4)	75 487 (19.3)	331 743 (19.5)	36 541 (19.3)	166 375 (19.5)
3	57 338 (18.7)	258 673 (18.9)	68 097 (18.2)	292 211 (18.5)	72 871 (18.6)	319 030 (18.8)	34 954 (18.5)	159 514 (18.7)
4	65 794 (21.5)	289 161 (21.2)	79 136 (21.2)	327 264 (20.7)	82 072 (20.9)	352 652 (20.8)	39 850 (21.0)	177 029 (20.8)
5, most deprived	64 581 (21.1)	273 330 (20.0)	82 940 (22.2)	322 995 (20.4)	78 921 (20.1)	329 937 (19.4)	37 693 (19.9)	164 058 (19.3)
Missing	973 (0.3)	4311 (0.3)	1525 (0.4)	6035 (0.4)	1088 (0.3)	4742 (0.3)	633 (0.3)	2821 (0.3)

Data are n (column %) unless otherwise specified.

* GP consultations in the year before cohort entry.

BMI, body mass index; IMD, Index of Multiple Deprivation.

Sex distribution was broadly balanced in most cohorts, ranging from 49% to 57% female. However, in the UTI cohort women comprised approximately 80% of participants. Median age ranged from 42 years (IQR 30–60) in the meningitis/encephalitis cohort to 73 (IQR 59–83) in the sepsis cohort. Across all cohorts, compared with matched comparators, individuals with infection had more comorbidities (based on CCI), were more deprived, and had higher rates of smoking, obesity and harmful alcohol use. Individuals with and without infection were similar in terms of deprivation across all cohorts (individual level IMD was missing and supplemented with practice-level data for approximately 20% of individuals across infection cohort).

Individuals included in complete-case analyses (implicitly adjusted for age, sex, calendar period through matching and explicitly adjusted for IMD quintile, lifestyle covariates, comorbidities and ethnicity) were similar with respect to sex for UTI and more likely to be male in the GE, LRTI and meningitis/encephalitis cohorts compared with those with no missing data (online supplemental table 2).

### Main analysis

**Figure 1:**
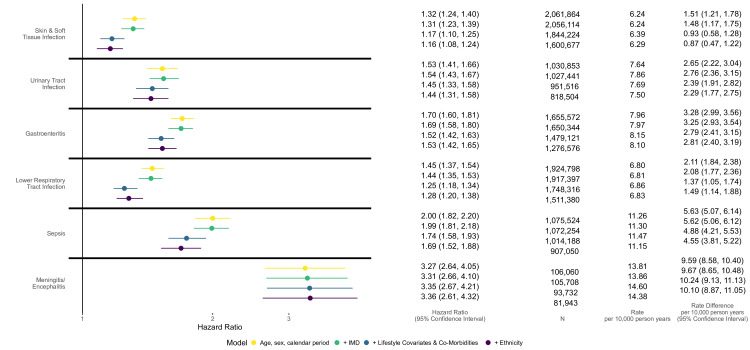
HRs (95% CI) comparing hazards of SMI in people with each of the six study infections to a matched population without the infection. Fitted to individuals with complete data for all variables included in each model and from valid matched sets (matched sets including one infection-exposed individuals and at least one unexposed comparator without infection). Number of individuals (n) and absolute rate per 10000 person-years are given for people with infection. The rate difference per 10000 person-years (with 95% CIs) between people with and without infection is calculated as the rate in those with infection minus the estimated rate in those without infection (rate in those with infection × (1/HR)). IMD, Index of Multiple Deprivation; SMI, severe mental illness.

Rate of SMI varied across infection cohorts, ranging from 5.15 per 10 000 person-years (95% CI 5.02 to 5.27) in the LRTI cohort to 6.31 (95% CI 5.74 to 6.93)in the meningitis/encephalitis cohort . After implicit adjustment for matching variables (age, sex, general practice, calendar period) and explicit adjustment for other potential confounders (deprivation, smoking status, harmful alcohol use, obesity, chronic comorbidities, ethnicity) there was evidence of an association between all infections and increased hazards of incident SMI (figure 1), with an attenuation of HRs after sequential adjustment. In fully adjusted models, there was a particularly strong association for the positive control exposure meningitis/encephalitis HR 3.36 (95% CI 2.61 to 4.32). HRs for other infections ranged from 1.16 (95% CI 1.08 to 1.24) for SSTI, to 1.69 (95% CI 1.52 to 1.88) for sepsis; with HR 1.44 (95% CI 1.31 to 1.58) for UTI, 1.53 (95% CI 1.42 to 1.65) for GE and 1.28 (95% CI 1.20 to 1.38) for LRTI. The fully adjusted SMI rate difference between those with and without infection ranged from 0.87 per 10 000 person years (95% CI 0.47 to 1.22) in the SSTI cohort to 10.10 (95% CI 8.87 to 11.05) in the meningitis/encephalitis cohort (online supplemental table 3).

### Sensitivity analyses

The results of most sensitivity analyses were broadly consistent with those of the main analyses (online supplemental table 4). When restricting to individuals with at least one general practitioner (GP) consultation in the year before cohort entry a very slight reduction in HRs was seen in all cohorts except the meningitis/encephalitis cohort; however, CIs included the main analysis estimates in all cases. Effect estimates were slightly attenuated when we started follow-up 1 year post-infection, especially in the sepsis and meningitis/encephalitis cohorts. Ending the study on 1 March 2020, to investigate any effect of including pandemic time in our main analyses, gave very similar results in all cohorts including the LRTI cohort (HR 1.26 (95% CI 1.16 to 1.37) compared with HR 1.28 (95% CI 1.20 to 1.38) in the main analysis). Using a missing category approach to ethnicity yielded similar results to the main complete case analyses in all cohorts.

### Secondary analyses

#### Temporal association

The association between infections and incident SMI attenuated over time but remained elevated up to 5 years following infection across all infection types (online supplemental table 5). The highest risk was observed in the first 6 months post-infection ranging from HR 1.46 (95% CI 1.17 to 1.82) for SSTI to HR 8.73 (95% CI: 4.89 to 15.58) for meningitis/encephalitis. By 5 years post-infection, HRs remained elevated although reduced in magnitude; for example, HR 1.19 (95% CI 1.09 to 1.30) for SSTI and HR: 3.93 (95% CI 2.95 to 5.23) for meningitis/encephalitis.

#### Infection severity and antimicrobial treatment

**Figure 2:**
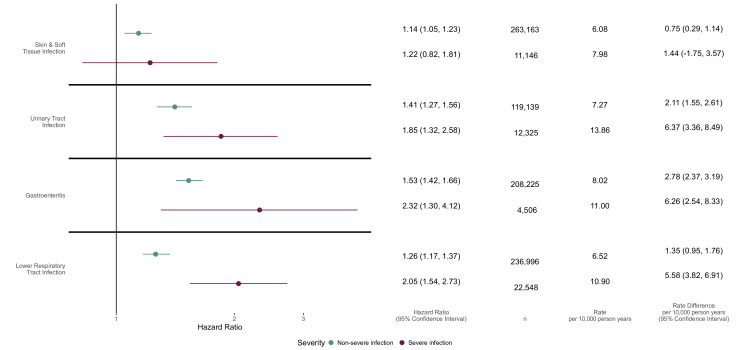
HRs (95% CI) comparing hazards of SMI in people with severe or non-severe infections to a matched population without the specific infection. Fitted to individuals with complete data for all variables included in the fully adjusted model (age, sex, IMD, lifestyle covariates, CCI and ethnicity) and from valid matched sets (matched sets including one infection-exposed individuals and at least one unexposed comparator without infection). Number of individuals (n) and absolute rate per 10000 person-years are given for people with infection. The rate difference per 10000 person-years (with 95% CIs) between people with and without infection is calculated as the rate in those with infection minus the estimated rate in those without infection (rate in those with infection × (1/HR)). CCI, Charlson Comorbidity Index; IMD, Index of Multiple Deprivation; SMI, severe mental illness.

While point estimates were higher for all severe infections compared with non-severe infections, CIs overlapped in all cohorts, except LRTI (figure 2).

Among infections without a record of antimicrobial prescription, the HRs for SMI were higher compared with infections with recorded antimicrobial treatment, although CIs overlapped in all cohorts except for UTI (those without a recorded antimicrobial prescription: HR 1.62 (95% CI 1.43 to 1.84); those with: HR 1.24 (95% CI 1.13 to 1.35)) (online supplemental table 6).

#### Effect modification

Across all infections, there was no evidence of effect modification by age or sex. In the LRTI cohort, there was a stronger association among those with moderate or severe frailty compared with those who were fit (online supplemental table 7).

## Discussion

In this large, population-based, matched cohort study using routinely collected primary care data, SMI was increased in individuals following infection compared with those without, regardless of infection type. HRs ranged from 1.16 for SSTI to 3.36 for meningitis/encephalitis (positive control exposure), with a higher risk of SMI following the more severe infections sepsis and meningitis/encephalitis. Point estimates were higher for hospitalised infections compared with those recorded in primary care alone, although CIs overlapped for all except LRTI. Associations were strongest in the year post-infection but persisted up to 5 years. Results were supported across a series of sensitivity analyses.

### In context with existing literature

Prior research on acute infections and incident SMI risk has largely focused on severe infections recorded in hospital data, typically occurring in childhood or adolescence. Around half of previous studies have focused on CNS infections alone.^8^ We are unaware of prior studies investigating associations between a range of acute infections recorded in primary care in adults and incident SMI risk.

In our study, we saw associations between GE, UTI, LRTI, and SSTI diagnosed in adults in primary care and risk of incident SMI. Findings were consistent with a study of intestinal infectious diseases among individuals of mixed ages in Taiwan, in which infections recorded in health insurance data from both inpatient and outpatient settings were associated with both schizophrenia and bipolar disorder risk.^19^ A Danish population-based study showed elevated schizophrenia risks of similar magnitude to those seen in our study associated with infections affecting a range of sites including urogenital infections (HR 1.90, 95% CI 1.79 to 2.01), respiratory infections (HR 1.53, 95% CI 1.46 to 1.61) and skin infections (HR 1.71, 95% CI 1.62 to 1.80).^20^

Our finding of a stronger association between sepsis and incident SMI risk than between the more acute infections and SMI is consistent with prior studies, including the Danish study showing a nearly twofold increase in schizophrenia risk associated with sepsis.^20^ Persistent psychiatric morbidity, including anxiety, depression, and post-traumatic stress disorder, as well as cognitive impairment and functional disability have been shown in studies of sepsis survivors.^21 22^

Our positive control exposure, meningitis/encephalitis showed the strongest association with incident SMI risk, with our HR of 3.36 in line with that of a previous UK study showing a similar psychosis risk, and an even greater elevation in the risk of bipolar disorder, after encephalitis in adults.^11^ The effect size we saw in adults was stronger than in previous studies of CNS infections in children and psychosis risk (OR 1.68, 95% CI 1.08 to 2.62).^8^

We have recently reported broadly similar associations between common, acute infections and incident anxiety/depression,^7^ except meningitis/encephalitis which had a lower reported risk of anxiety/depression (HR 1.65, 95% CI 1.58 to 1.73) than our SMI results (HR 3.36), in line with Granerod *et al.*^11^

Immune-brain interactions including direct neurotoxicity, alterations to blood-brain barrier permeability leading to CNS inflammation and disturbances to gut microbiota have been proposed as potential mechanisms to explain associations between infections affecting a range of body sites and SMI risk.^23 24^

In our study, SMI risk peaked in the first 6 months post-infection (ranging from HR 1.46 for SSTI to 8.73 for meningitis/encephalitis), attenuating yet remaining elevated at 5 years (HRs 1.19–3.93), with higher risk closer to when the infection occurred. This finding is consistent with Danish cohorts showing that the risks of mental health conditions were highest shortly after exposure to infections, although as in our study, effects persisted longer-term (up to 10 years).^25^

### Strengths and limitations

A key strength of our study is its large, nationally representative adult population drawn from primary care electronic health records, enabling comprehensive capture of infection exposures and subsequent SMI diagnoses over extended follow-up. To our knowledge, this is one of the first studies to examine such a broad spectrum of acute infections in adults in relation to incident SMI, using a robust matched cohort design while adjusting for important confounders.

However, there are limitations. Individuals experiencing some infections (particularly more common and mild infections) may not attend primary care for their symptoms. Consequently, it is likely that some of the individuals included in cohorts as comparators may have had the infection of interest. This misclassification of individuals with infection as uninfected, if unrelated to SMI status, would only bias results towards the null, meaning our results would underestimate the true association. However, if health-seeking behaviour for infection symptoms is influenced by underlying mental health status (ie, those with undiagnosed SMI could be more or less likely to see their GP with infection symptoms) our effect estimates could be underestimates or overestimates depending on the unpredictable relationship between SMI status and infection-related health seeking behaviour.

We relied on primary care data for outcome capture, as we did not have access to data from specialist mental health settings, which may have resulted in some SMI episodes being missed or the incidence recording date being delayed. Nevertheless, all admissions with an incident SMI diagnosis should result in a GP record, although with potential for a short time lag. Missed or diagnosed late SMI events are expected to occur at a similar rate between uninfected and infected individuals, limiting the potential for bias. Previous research has reported primary care data to be a valid and suitable data source for research into incident SMI diagnoses as well as for assessing the prevalence of SMI in England.^26 27^ The Quality and Outcomes Framework introduced in 2004, prior to our study start, financially incentivises UK practices to keep a register of individuals living with SMI, improving recording.^28^

Individuals living with undiagnosed SMI may have more infections: pre-existing SMI affects the presence and severity of acute infections.^29^ This could have resulted in some reverse causation in our study, although sensitivity analysis starting follow-up 1 year after infection still demonstrated an association, although with reduced effect estimates (particularly for sepsis and meningitis/encephalitis). This may provide evidence of an association waning over time. It may also reflect delayed recording in primary care of infections likely to have been managed in hospital settings. For example, it is possible that true infection dates for sepsis and meningitis/encephalitis happened some time earlier than the primary care record indicated (due to primary care recording following hospital admission for the infection), leading to a greater attenuation of effect when follow-up started 1 year after primary care recorded infection date.

Limitations intrinsic to primary care data include unmeasured confounders (eg, genetic risk factors for SMI that might also impact the immune response to infections), which may influence outcomes despite rigorous confounder adjustment.

Using complete-case analyses, excluding individuals with missing covariate data, may have introduced selection bias. However, consistent results of sequential models suggest that this is unlikely to be a considerable problem. We also saw consistent results repeating our analysis using a missing category approach to handling missing ethnicity data. In addition, the characteristics of the study population excluded due to missing data were similar to those included in the complete-case analysis. Finally, the numbers of SMI diagnoses were relatively limited, leading to reduced power for some secondary analyses (eg, effect modification by infection severity where CIs were wide).

Whether there is evidence of a stronger association among infections without an accompanying antimicrobial prescription record, potentially due to more severe infections receiving hospital treatment, requires further investigation. Clinically, these findings emphasise the importance of prompt infection diagnosis and treatment and comprehensive preventive strategies including vaccination. In addition, mental health monitoring after severe infections requiring critical care should consider SMI risk.^30^ Future prospective, longitudinal studies are essential to disentangle mechanisms and identify targeted interventions to further mitigate infection associated SMI risk.

## Conclusion

In conclusion, our study findings suggest SMI risk is higher among adults with acute infections, compared with those without, especially for more severe infections including sepsis and meningitis/encephalitis. Our study underscores the importance of infection prevention, early treatment and monitoring for mental health symptoms following severe infection episodes. Providing timely infection treatment, targeted mental health risk management, and where appropriate offering relevant vaccinations, may limit incident SMI.

## Supplementary material

10.1136/bmjment-2026-302640 online supplemental file 1

## Data Availability

No data are available.
