# Supplementary material for: Infections and severe mental illness: a population-based matched cohort study
Source: BMJ Ment Health. 2026 May 27;29(1):e302640. doi: 10.1136/bmjment-2026-302640 (PMC13218188; doi:10.1136/bmjment-2026-302640)
Supplement: online supplemental file 1 [file bmjment-29-1-s001.docx]

**Supplementary file for Infections and Severe Mental Illness: A Population-Based Matched Cohort Study**


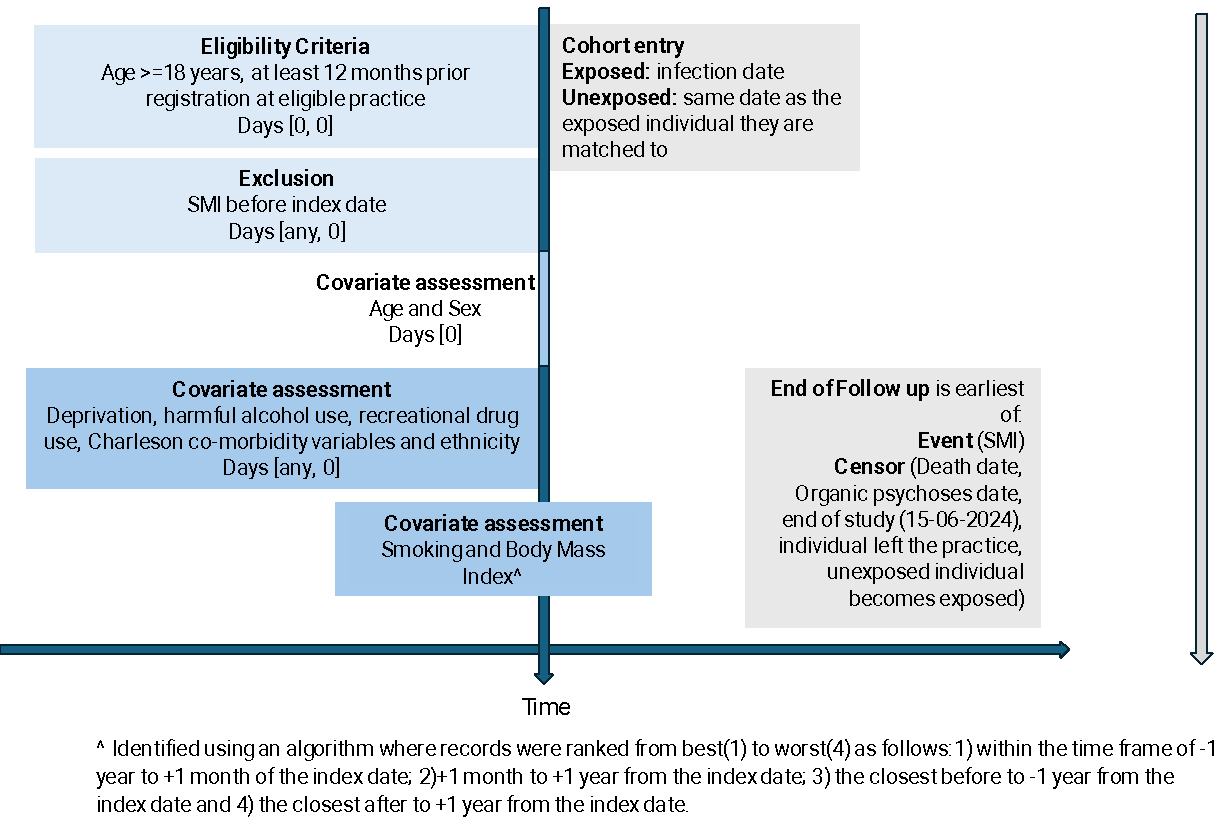


**Supplementary figure 1:** Illustration of study design, describing eligibility criteria, covariate ascertainment and follow-up.

**
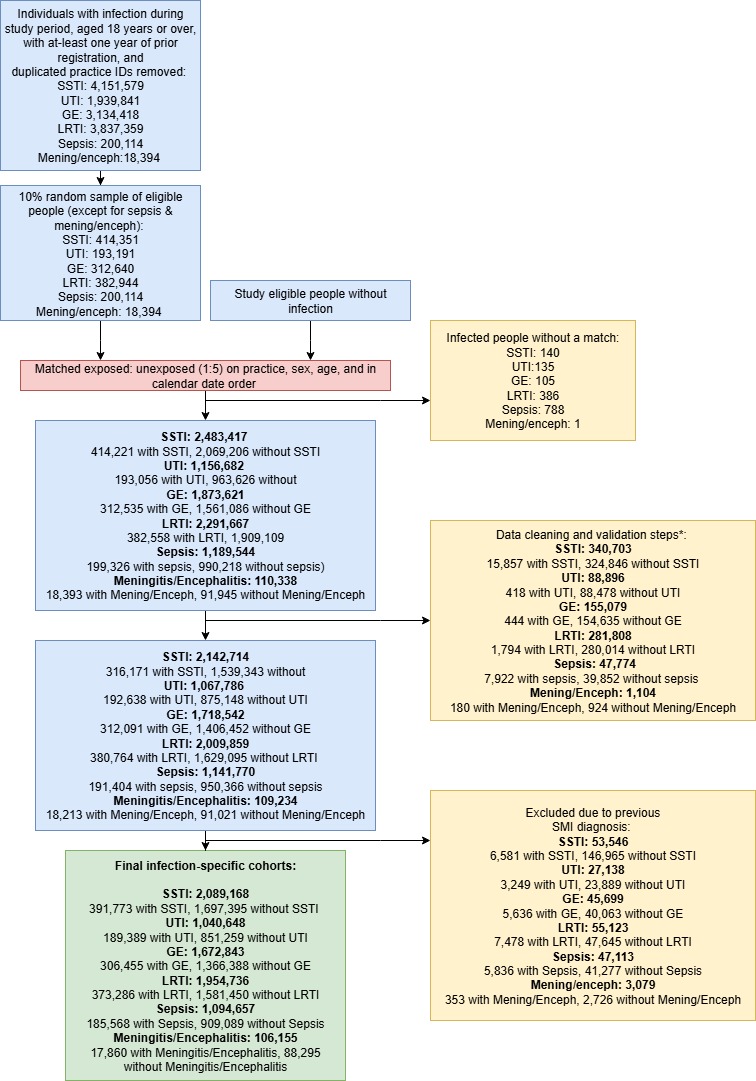
**

**Supplementary figure 2:** Overview of how cohorts were defined for each infection of interest. SSTI = Skin and soft tissue infection, UTI = Urinary tract infection, GE = Gastroenteritis, LRTI = Lower respiratory tract infection. *Supplementary methods 1 for more detail on data cleaning & validation

**Supplementary methods 1:** Data cleaning and validation description

Due to the very large sample sizes of eligible individuals with records of common infections (i.e., GE (n=3,134,418), LRTI (n=3,837,359), SSTI (n=4,151,579) and UTI (n=1,939,841)), it was not computationally possible to run the matching algorithm for cohorts of these sizes. We therefore took a random 10% sample of infection-exposed individuals, stratified by practice, prior to matching. Matching was then conducted with the infection-exposed individuals not selected in the 10% random sample eligible for inclusion as potential comparators. These individuals could act as comparators up to their infection date, at which point they were censored. When potential comparators had a record of infection prior to matching, they were dropped as a comparator. All infection-exposed individuals were included in the sepsis and meningitis/encephalitis cohorts.

**Supplementary Table 1:** Definition of covariates

| **Harmful alcohol use** | Harmful alcohol use was defined based on primary care morbidity codes suggesting harmful or heavy alcohol use (including alcohol dependency codes and codes related to physical/psychological harm related to alcohol use) or a prescription for drugs used to maintain abstinence. Individuals were defined as harmful alcohol users on the date of the first record of a relevant morbidity code or prescription. |
| --- | --- |
| **Smoking status and obesity** | Data on **smoking status** and **being obese** (i.e., body mass index [BMI] suggesting individuals are obese) were obtained from primary care records for these measures, using the status recorded closest to index date, based on a previously defined algorithm, where records identified within −1 year to +1 month of the index date were regarded as the best, +1 month to +1 year from the index date as second best, the nearest before −1 year from the index date as the third best, and the nearest after +1 year from the index date as the worst. BMI was calculated from height and weight measurements, and we defined a binary obese variable based on a BMI of 30 kg/m2 or more.^1^ |
| **Co-morbidities** | Co-morbidities were defined using individual diseases (identified using primary care codes) used for the Charlson comorbidity index (CCI).^2^  Comorbidities included and their assigned weights: 1) weighted 1: myocardial infarction, congestive heart failure, peripheral vascular disease, cerebrovascular disease, dementia, lung disease, rheumatoid diseases, peptic ulcer, mild liver disease, diabetes without complications; 2) weighted 2: diabetes with complications, hemiplegia, chronic kidney disease, cancer including lymphoma; 3) weighted 3: moderate/severe liver disease; and 4) weighted 6: metastatic cancer and HIV.  Individuals were regarded as having one of these diagnoses from the earliest record of a relevant diagnostic code. An individual’s total score was calculated and categorised into low (0), moderate (1-2), or severe (3 or more). |
| **Ethnicity** | Ethnicity was identified based on a previously validated algorithm using primary care records.^3^ And classified as: |
| **Frailty** | In a secondary analysis, we used the electronic frailty index (eFrailty) as a measure of **frailty.^4^** The eFrailty index uses primary care morbidity coding across 36 deficits (deficits include specific morbidities [e.g., arthritis, hypertension, Parkinsonism], symptoms [e.g., activity limitation, dizziness], social issues [e.g., vulnerability, care requirements]) to categorise individuals as fit, mildly frail, moderately frail, or severely frail. The index uses a cumulative model of equally weighted individual deficits to calculate a score between zero and one based on the proportion of deficits identified out of the full list of thirty-six.^4^ |

|  | **Gastroenteritis** | | **Lower respiratory tract infection** | | **Skin and soft tissue infection** | | **Urinary tract infection** | | **Sepsis** | | **Meningitis/encephalitis** | |
| --- | --- | --- | --- | --- | --- | --- | --- | --- | --- | --- | --- | --- |
|  | **Included*** | **Excluded**** | **Included** | **Excluded** | **Included** | **Excluded** | **Included** | **Excluded** | **Included** | **Excluded** | **Included** | **Excluded** |
| **Total**  **N (%)** | 1,290,897 (77.2) | 381,946 (22.8) | 1,536,600 (78.6) | 418,136 (21.4) | 1,623,795  (77.7) | 465,373  (22.3) | 826,510  (79.4) | 214,138  (20.6) | 923,236  (84.3) | 171,421  (15.7) | 82,016  (77.3) | 24,139  (22.7) |
| **Median age (IQR) years** | 52  (35,69) | 41  (26,67) | 57  (42,71) | 52 (33,74) | 52  (37,67) | 42  (27,65) | 56 (36,72) | 53  (29, 78) | 73 (60,82) | 74 (54,85) | 45  (32,62) | 33  (24,50) |
| **Age** |  |  |  |  |  |  |  |  |  |  |  |  |
| **18-29** | 122,852 (32.2%) | 206,475 (16.0%) | 81,545 (19.5%) | 137,494 (8.9%) | 225,428 (13.9%) | 140,183 (30.1%) | 130,468 (15.8%) | 54,701 (25.5%) | 26,654 (2.9%) | 13,828 (8.1%) | 15,773 (19.2%) | 9,659 (40.0%) |
| **30-39** | 60,993 (16.0%) | 196,797 (15.2%) | 60,490 (14.5%) | 196,947 (12.8%) | 247,890 (15.3%) | 75,346 (16.2%) | 111,765 (13.5%) | 26,171 (12.2%) | 40,678 (4.4%) | 10,559 (6.2%) | 17,332 (21.1%) | 5,451 (22.6%) |
| **40-59** | 79,415 (20.8%) | 383,998 (29.7%) | 104,791 (25.1%) | 508,755 (33.1%) | 538,915 (33.2%) | 110,755 (23.8%) | 215,655 (26.1%) | 38,564 (18.0%) | 159,192 (17.2%) | 27,235 (15.9%) | 25,883 (31.6%) | 5,130 (21.3%) |
| **60+** | 118,686 (31.1%) | 503,627 (39.0%) | 171,310 (41.0%) | 693,404 (45.1%) | 611,562 (37.7%) | 139,089 (29.9%) | 368,622 (44.6%) | 94,702 (44.2%) | 696,712 (75.5%) | 119,799 (69.9%) | 23,028 (28.1%) | 3,899 (16.2%) |
| **Sex** |  |  |  |  |  |  |  |  |  |  |  |  |
| **Female** | 186,041 (48.7%) | 773,098 (59.9%) | 205,203 (49.1%) | 891,043 (58.0%) | 945,767 (58.2%) | 217,285 (46.7%) | 657,887 (79.6%) | 168,509 (78.7%) | 454,100 (49.2%) | 80,769 (47.1%) | 48,756 (59.4%) | 10,616 (44.0%) |
| **Male** | 195,905 (51.3%) | 517,799 (40.1%) | 212,933 (50.9%) | 645,557 (42.0%) | 678,028 (41.8%) | 248,088 (53.3%) | 168,623 (20.4%) | 45,629 (21.3%) | 469,136 (50.8%) | 90,652 (52.9%) | 33,260 (40.6%) | 13,523 (56.0%) |

**Supplementary Table 2:** Age and sex of individuals included and excluded from the complete case analyses (i.e., final fully adjusted model implicitly adjusting for age, sex, calendar period, and explicitly deprivation, lifestyle covariates and co-morbidities, and ethnicity).

*****Included: complete case analysis sample **Excluded due to missing data

**Supplementary Table 3**: **Absolute incidence rates and rate differences of common mental disorder in infection cohorts**

| **Model** |  | **Gastroenteritis** | **Lower respiratory tract infection** | **Skin and soft tissue infection** | **Urinary tract infection** | **Sepsis** | **Meningitis/**  **encephalitis** |  |
| --- | --- | --- | --- | --- | --- | --- | --- | --- |
| 1. **Age, sex, calendar period** | **N*** | 1,672,552 | 1,924,798 | 2,061,864 | 1,038,531 | 1,075,524 | 106,060 |  |
|  | **Person-years*** | 2,059,400 | 2,606,371 | 2,750,529 | 1,255,914 | 603,732 | 111,555 |  |
|  | **SMI events*** | 1,641 | 1,771 | 1,715 | 959 | 680 | 154 |  |
|  | **Rate*^†^** | 7.96 | 6.80 | 6.24 | 7.64 | 11.26 | 13.81 |  |
|  | **Rate difference (95% CI)**†****** | 3.28 (2.99, 3.56) | 2.11 (1.84, 2.38) | 1.51 (1.21, 1.78) | 2.65 (2.22, 3.04) | 5.63 (5.07, 6.14) | 9.59 (8.58, 10.40) |  |
| 1. **+ IMD** | **N*** | 1,650,344 | 1,917,397 | 2,056,114 | 1,027,441 | 1,072,254 | 105,708 |  |
|  | **Person-years*** | 2,052,620 | 2,594,230 | 2,742,744 | 1,251,676 | 601,785 | 111,140 |  |
|  | **SMI events*** | 1635 | 1767 | 1712 | 956 | 680 | 154 |  |
|  | **Rate*^†^** | 7.97 | 6.81 | 6.24 | 7.86 | 11.30 | 13.86 |  |
|  | **Rate difference (95% CI)**** | 3.25 (2.93, 3.54) | 2.08 (1.77, 2.36) | 1.48 (1.17, 1.75) | 2.76 (2.36, 3.15) | 5.62 (5.06, 6.12) | 9.67 (8.65, 10.48) |  |
| 1. **+ Lifestyle covariates** | **N*** | 1,479,121 | 1,748,316 | 1,844,224 | 951,516 | 1,014,188 | 93,732 |  |
|  | **Person-years*** | 1,951,587 | 2,504,180 | 2,601,982 | 1,215,653 | 581,770 | 104,129 |  |
|  | **SMI events*** | 1,590 | 1,717 | 1,663 | 935 | 667 | 152 |  |
|  | **Rate*^†^** | 8.15 | 6.86 | 6.39 | 7.69 | 11.47 | 14.60 |  |
|  | **Rate difference (95% CI)**†****** | 2.79 (2.41, 3.15) | 1.37 (1.05, 1.74) | 0.93 (0.58, 1.28) | 2.39 (1.91, 2.82) | 4.88 (4.21, 5.53) | 10.24 (9.13, 11.13) |  |
| 1. **+ Ethnicity** | **N*** | 1,290,690 | 1,511,380 | 1,600,677 | 818,504 | 907,050 | 81,943 |  |
|  | **Person-years*** | 1,766,163 | 2,284,547 | 2,361,067 | 1,099,453 | 529,154 | 93,859 |  |
|  | **SMI events*** | 1,430 | 1,561 | 1,484 | 824 | 590 | 135 |  |
|  | **Rate*^†^** | 8.10 | 6.83 | 6.29 | 7.50 | 11.15 | 14.38 |  |
|  | **Rate difference (95% CI)**†****** | 2.81 (2.40, 3.19) | 1.49 (1.14, 1.88) | 0.87 (0.47, 1.22) | 2.29 (1.77, 2.75) | 4.55 (3.8  1, 5.22) | 10.10 (8.87, 11.05) |  |
| *Number of people, person-years, SMI events, and rate in people with infection. † Rate per 10,000 person-years. **SMI rate difference between those with and without infection. | | | | | | | | |

**Supplementary Table 4:** Results of sensitivity analyses

|  | **Analysis** | **Description and justification** | **HR (95% CI)** | | | | | |
| --- | --- | --- | --- | --- | --- | --- | --- | --- |
|  |  |  | **Gastroenteritis** | **Lower respiratory tract infection** | **Skin and soft tissue infection** | **Urinary tract infection** | **Sepsis** | **Meningitis/**  **encephalitis** |
|  | **Main analysis (fully adjusted model)** | **Included for comparison** | **1.53 (1.42, 1.65)** | **1.28 (1.20, 1.38)** | **1.16 (1.08,1.24)** | **1.44 (1.31, 1.58)** | **1.69 (1.52, 1.88)** | **3.36 (2.61, 4.32)** |
| 1 | Restricting to individuals with >=1 consultation with their GP in the year before cohort entry | To exclude practice non-attenders | 1.51 (1.40, 1.62) | 1.26 (1.17, 1.35) | 1.13 (1.06,1.22) | 1.43 (1.30, 1.57) | 1.68 (1.50, 1.87) | 3.45(2.67, 4.46) |
| 2 | Main analysis without censoring organic psychoses diagnoses | To avoid missing outcomes | 1.53 (1.42, 1.64) | 1.28 (1.20, 1.38) | 1.16 (1.08, 1.24) | 1.44 (1.32, 1.58) | 1.69 (1.52, 1.89) | 3.39 (2.64, 4.35) |
| 3 | Main analysis starting follow-up 1 year after initial infection, excluding those with SMI in the year between index date and start of follow-up | To limit outcome ascertainment bias or reverse causality as a potential explanation for our findings | 1.49 (1.38, 1.62) | 1.24 (1.15, 1.34) | 1.08 (1.00,1.17) | 1.38 (1.24, 1.53) | 1.48 (1.30,1.68) | 2.28 (1.66, 3.14) |
| 4 | In SSTI cohort, additionally adjusting for injecting drug use | Injecting drug use predisposes to SSTI and is also associated with SMI so control for as an additional confounder | N/A | N/A | 1.15 (1.07,1.23) | N/A | N/A | N/A |
| 5 | Repeat analyses ending study on 1^st^ March 2020 | Our results may be biased by changes in consulting practices due to the pandemic. We will repeat our analysis ending the study on 1st March 2020 to explore the impact of including pandemic time in our analyses. | 1.53 (1.40, 1.67) | 1.26 (1.16, 1.37) | 1.19 (1.10,1.30) | 1.39 (1.25,1.55) | 1.54 (1.33, 1.79) | 3.75 (2.74, 5.13) |
| 6 | Missing category approach to ethnicity | To investigate the effect of missing ethnicity data | 1.53 (1.43, 1.64) | 1.25 (1.17, 1.34) | 1.17 (1.10, 1.26) | 1.46 (1.34, 1.59) | 1.74 (1.57, 1.93) | 3.38 (2.69, 4.26) |

**Supplementary Table 5:** Association between each infection and SMI over time, HR (95%CI)

|  | **Gastroenteritis** | **Lower respiratory tract infection** | **Skin and soft tissue infection** | **Urinary tract infection** | **Sepsis** | **Meningitis/**  **Encephalitis** |
| --- | --- | --- | --- | --- | --- | --- |
| **Main analyses:  for comparison, fully adjusted model** | **1.53 (1.42, 1.65)** | **1.28 (1.20, 1.38)** | **1.16 (1.08,1.24)** | **1.44 (1.31, 1.58)** | **1.69 (1.52, 1.88)** | **3.36 (2.61, 4.32)** |
| 0-6 months | 1.72 (1.37, 2.16) | 1.53 (1.24, 1.90) | 1.46 (1.17, 1.82) | 1.84 (1.39, 2.45) | 2.24 (1.70, 2.94) | 8.73 (4.89, 15.58) |
| 0-1 year | 1.65 (1.40, 1.99) | 1.43 (1.22, 1.67) | 1.50 (1.28, 1.76) | 1.60 (1.30, 1.98) | 2.30 (1.87, 2.82) | 6.94 (4.42, 10.89) |
| 0-2 years | 1.67 (1.47, 1.90) | 1.38 (1.23, 1.56) | 1.30 (1.15, 1.47) | 1.54 (1.31, 1.80) | 1.94 (1.66, 2.28) | 4.69 (3.30, 6.67) |
| 0-3 years | 1.58 (1.42, 1.76) | 1.27 (1.15, 1.41) | 1.22 (1.10, 1.36) | 1.54 (1.34, 1.77) | 1.88 (1.63, 2.16) | 3.97 (2.88, 5.47) |
| 0-4 years | 1.58 (1.43, 1.74) | 1.30 (1.19, 1.43) | 1.21 (1.10, 1.32) | 1.55 (1.37, 1.75) | 1.81 (1.60, 2.06) | 3.92 (2.92, 5.27) |
| 0-5 years | 1.60 (1.46, 1.75) | 1.25 (1.15, 1.37) | 1.19 (1.09, 1.30) | 1.50 (1.34, 1.68) | 1.76 (1.56 (1.98) | 3.93 (2.95, 5.23) |

**Supplementary Table 6:** Secondary analysis: Investigating the role of antimicrobial prescriptions, HR (95% CI)

|  |  | **Gastroenteritis** | **Lower respiratory tract infection** | **Skin and soft tissue infection** | **Urinary tract infection** |
| --- | --- | --- | --- | --- | --- |
| **Main analyses (for comparison), fully adjusted model)** |  | **1.53 (1.42,1.65)** | **1.28 (1.20, 1.38)** | **1.16 (1.08,1.24)** | **1.44 (1.31, 1.58)** |
| Infections **with** a record of antimicrobial prescription within 7 days before or after infection | HR (95% CI) | 1.19 (0.94,1.50) | 1.20 (1.12, 1.28) | 1.14 (1.06, 1.23) | 1.24 (1.13, 1.35) |
|  | Total n; SMI events | 16,133; 81 | 263,141; 1,293 | 191,625; 889 | 105,146; 559 |
| Infections **without** a record of antimicrobial prescription within 7 days before or after infection | HR (95% CI) | 1.55 (1.46, 1.66) | 1.35 (1.21, 1.49) | 1.23 (1.13, 1.34) | 1.62 (1.43, 1.84) |
|  | Total n; SMI events | 261,539; 1,465 | 101,870; 454 | 117,069; 565 | 47,619; 260 |

**Supplementary Table 7**: Secondary analyses: Investigating age, sex, and frailty as effect modifiers, HR (95% CI)

|  | **Gastroenteritis** | **Lower respiratory tract infection** | **Skin and soft tissue infection** | **Urinary tract infection** | **Sepsis** | **Meningitis/**  **encephalitis** |
| --- | --- | --- | --- | --- | --- | --- |
| **Age** |  |  |  |  |  |  |
| 18-29 | 1.40 (1.19, 1.65) | 1.58 (1.31, 1.89) | 1.19 (1.02, 1.39) | 1.49 (1.20, 1.83) | 1.63 (1.10, 2.43) | 3.18 (1.94, 5.23) |
| 30-39 | 1.60 (1.35, 1.91) | 1.45 (1.23, 1.71) | 1.20 (1.01, 1.41) | 1.06 (0.83, 1.36) | 2.13(1.52, 2.99) | 2.29 (1.32, 3.98) |
| 40-59 | 1.67 (1.47, 1.90) | 1.38 (1.22, 1.55) | 1.18 (1.05,1.34) | 1.51 (1.28, 1.79) | 1.99 (1.60, 2.48) | 3.40 (2.18, 5.32) |
| 60+ | 1.45 (1.27, 1.65) | 1.04 (0.93, 1.18) | 1.10 (0.97, 1.24) | 1.53 (1.32, 1.78) | 1.53 (1.33, 1.76) | 5.01 (2.94, 8.53) |
| **Sex** |  |  |  |  |  |  |
| Male | 1.52 (1.40, 1.69) | 1.28 (1.14, 1.43) | 1.22 (1.09, 1.36) | 1.61 (1.28, 2.02) | 1.79 (1.52, 2.11) | 2.88 (1.83, 4.52) |
| Female | 1.54 (1.35, 1.71) | 1.29 (1.18, 1.41) | 1.12 (1.03, 1.23) | 1.41 (1.27, 1.56) | 1.62 (1.41, 1.87) | 3.60 (2.67, 4.87) |
| **Frailty (restricting study population to 65-95-year-olds)** |  |  |  |  |  |  |
| Fit (<=0.12) | 0.95 (0.71, 1.27) | 0.76 (0.59, 0.98) | 1.08 (0.83, 1.39) | 1.02 (0.72, 1.44) | 0.92 (0.57, 1.49) | 2.59 (0.64, 10.54) |
| Mild frailty (0.13-0.24) | 1.67 (1.33, 2.11) | 1.19 (0.96, 1.46) | 0.99 (0.80, 1.24) | 1.52 (1.16, 1.99) | 1.55 (1.19, 2.02) | 7.39 (2.49, 21.91) |
| Moderate frailty (0.25-0.36) | 1.53 (1.13, 2.06) | 1.42 (1.07, 1.89) | 1.07 (0.78, 1.46) | 1.65 (1.17, 2.32) | 1.51 (1.15, 1.99) | 5.06 (1.30, 19.68) |
| Severe frailty(>=0.37) | 2.34 (1.55, 3.53) | 1.98 (1.25, 3.13) | 1.99 (1.27, 3.11) | 2.25 (1.44, 3.51) | 1.76 (1.31, 2.36) | 9.29 (1.63, 52.90) |

**References:**

1. Bhaskaran K, Forbes HJ, Douglas I, Leon DA, Smeeth L. Representativeness and optimal use of body mass index (BMI) in the UK Clinical Practice Research Datalink (CPRD). *BMJ Open*. Sep 13 2013;3(9):e003389. doi:10.1136/bmjopen-2013-003389

2. Charlson ME, Carrozzino D, Guidi J, Patierno C. Charlson Comorbidity Index: A Critical Review of Clinimetric Properties. *Psychother Psychosom*. 2022;91(1):8-35. doi:10.1159/000521288

3. Mathur R, Bhaskaran K, Chaturvedi N, et al. Completeness and usability of ethnicity data in UK-based primary care and hospital databases. *J Public Health (Oxf)*. Dec 2014;36(4):684-92. doi:10.1093/pubmed/fdt116

4. Clegg A, Bates C, Young J, et al. Development and validation of an electronic frailty index using routine primary care electronic health record data. *Age Ageing*. May 2016;45(3):353-60. doi:10.1093/ageing/afw039
